# Timing of liver transplantation for pediatric acute liver failure due to mushroom poisoning: a case report and literature review

**DOI:** 10.1186/s12887-020-02249-9

**Published:** 2020-07-23

**Authors:** Chun-Feng Yang, Chu-Qiao Sheng, Yu Ao, Yu-Mei Li

**Affiliations:** Department of Pediatric Intensive Care Unit, The First Hospital of Jilin University, No. 1 Xinmin Street, 130021 Changchun, Jilin, China

**Keywords:** Pediatric acute liver failure, Liver transplantation, Mushroom poisoning, Prognostic model, Case report

## Abstract

**Background:**

Pediatric acute liver failure is a rare, life-threatening illness. Mushroom poisoning is a rare etiology. For patients with irreversible pediatric acute liver failure, liver transplantation is the ultimate lifesaving therapy. However, it is difficult to determine the optimal timing of transplantation. Here, we present a case of pediatric acute liver failure due to mushroom poisoning in northeastern China. He was treated with liver transplantation and recovered. To our knowledge, there are few reports about liver transplantation for pediatric acute liver failure caused by mushroom poisoning in mainland China.

**Case presentation:**

The patient was a previously healthy 9-year-old boy who gradually developed nausea, vomiting, jaundice and coma within 5 days after ingesting mushrooms. He was diagnosed with mushroom poisoning and acute liver failure. He was treated with conservative care but still deteriorated. On the 7th day after poisoning, he underwent LT due to grade IV hepatic encephalopathy. Twenty days later, he recovered and was discharged. A review of the literature revealed that the specific criteria and optimal timing of transplantation remain to be determined.

**Conclusions:**

Patients with pediatric acute liver failure should be transferred to a center with a transplant unit early. Once conservative treatment fails, liver transplantation should be performed.

## Background

Pediatric acute liver failure (PALF) is defined as severe impairment of liver function characterized by biochemical evidence of acute liver injury and coagulopathy, with an international normal ratio (INR) ≥ 2.0 and no evidence of chronic liver disease, diagnosed within 8 weeks from the onset of clinical symptoms. PALF can rapidly lead to multisystem organ failure with a fatal outcome [1, 2]. Some mushrooms, such as *Amanita phalloides*, are hepatotoxic and can cause PALF within 1–3 days after ingestion. The common therapies are primarily based on symptomatic and supportive care [3, 4]. Despite active treatment, some patients eventually die. Liver transplantation (LT) is an effective treatment for PALF. Without LT, PALF is a devastating process, with high mortality rate. With the advent and advancement of pediatric liver transplantation (PLT), patients are now likely to survive [5]. However, considering the spontaneous recovery potential and complications of PLT, the timing of PLT for PALF is very important.

The King’s College Hospital (KCH) criteria and Clichy criteria are the most commonly used prognostic models of acute liver failure (ALF) in adults. Among children, liver injury units (LIUs) and the Pediatric End-Stage Liver Disease (PELD) score are the most studied models [6]. Here, we present a case of PALF due to mushroom poisoning in northeastern China. To our knowledge, there are few reports about liver transplantation for pediatric acute liver failure caused by mushroom poisoning in mainland China. Whether these criteria are suitable for patients with PALF due to mushroom poisoning, in China has not been studied. This study preliminarily discusses this problem.

## Case presentation

A 9-year-old boy was transferred to our hospital due to jaundice and coma. He lived in a village in Jilin Province in northeastern China. His mother had collected wild mushrooms in the forest and cooked them for breakfast. The boy ate more mushrooms than the rest of the family. Approximately 12 h later, the boy and his parents presented to a local emergency department with nausea and vomiting. All of them were diagnosed with mushroom poisoning according to the history of mushroom ingestion. His parents’ symptoms gradually disappeared, and they were discharged the next day. However, symptoms in the boy gradually worsened even though gastric lavage had been performed. Twenty-four hours later, he developed abdominal pain, diarrhea and jaundice, so he was transferred to a tertiary hospital. On admission, he had jaundice. The laboratory data were as follows: alanine aminotransferase (ALT), 269 U/L (normal range: 9–50 U/L); total bilirubin (TBIL), 32 µmol/L (normal range: 6.8–30 µmol/L); direct bilirubin (DBIL), 11.6 µmol/L (normal range: 0.0-8.6 µmol/L); prothrombin time (PT), 24.3 s; INR, 2.08; Abdominal color Doppler ultrasound and abdominal computed tomography findings were normal. Virological analysis, autoantibody detection, and metabolic investigations were negative. He was diagnosed with mushroom poisoning based on medical history, clinical manifestations and exclusion of other causes. He also had ALF. He was treated with penicillin G, silybin, N-acetylcysteine and plasma exchange (the amount of plasma exchange was 40–50 ml/kg/d, once a day for 2 h each time). However, the patient was still deteriorating. On the 3rd day of admission, the patient began to suffer delirium and agitation. ALT increased to 4410 U/L, TBIL increased to 340 µmol/L and INR increased to 2.2. Ammonia was 73 µmol/L (normal range: 9–47 µmol/L). On the 5th day of admission, the patient became comatose, with a Glasgow Coma Scale (GCS) score of 3. Changes in liver function gradually resulted in a decrease in transaminase and an increase in bilirubin. The patient was transferred to our hospital for further management and an evaluation for PLT on the sixth day after mushroom ingestion.

Upon arrival at our institution, he had grade IV hepatic encephalopathy (HE). The laboratory data revealed that the PT was 62 s, and the INR was 5.5. ALT had decreased to 1200 U/L, TBIL had increased to 435 µmol/L. Ammonia was 126 µmol/L. Because of his worsening laboratory parameters and grade IV HE, LT was considered. ALF was one of the indications for LT, but contraindications still needed to be excluded. In particular, this patient had grade IV HE, and neurological sequelae were a worrisome problem. An electroencephalogram ( EEG ) showed that a diffuse δ wave pattern with a 2–3 Hz median amplitude was dominant, and a 4 Hzθ wave appeared after pain stimulation. Brain color Doppler ultrasonography showed no obvious intracranial hypertension. Cranial CT was normal. After consultation with the LT center, the patient was recommended to undergo PLT. With the consent of his parents and the approval of the ethical review board of Jilin Province, the patient underwent PLT on the 7th day after mushroom ingestion. The patient needed an emergency liver transplant, when the only source of liver was a living donor from a parent. The mother’s gastrointestinal symptoms disappeared within one day after eating mushrooms, and there was no other discomfort.Her liver function, renal function and abdomen CT were normal, and the other preoperative examinations were in accordance with the donor standard. The patient’s family agreed that the mother was the donor. The patient’ weight is 45 kg which is close to an adult’s weight. In order to ensure a reasonable graft to recipient weight ratio(GRWR), His mother donated the right lobe of her liver. The graft was implanted into the abdominal cavity using the piggyback technique. The histology of the explanted liver revealed massive hepatic necrosis compatible with severe cholestasis in residual hepatocyte. After operation, the patient returned to the pediatric intensive care unit with tracheal intubation and was placed on mechanical ventilation. Immunosuppression regimen included methylprednisolone combined with tacrolimus was started on the 2nd day after the operation. Prophylactic antibiotics including ganciclovir, meropenem and vancomycin, were administered. On the 2nd day after the operation, the patient’s consciousness gradually improved, and the GCS score increased to 11. On the 3rd day, his consciousness was clear, and the endotracheal tube was removed. Ammonia decreased to normal (34 µmol/L). One week later, liver function and coagulation were normal. Abdominal ultrasound didn’t show complications of hepatic vessels and biliary tract. Since there were no abnormal symptoms and signs of the nervous system, we did not recheck the brain imaging and EEG for him. The patient gradually improved and was discharged on postoperative day 20. So far, he has survived for 6 months without complications.

This report was approved by the ethics committee of The First Hospital of Jilin University, and informed consent was obtained from the patient’s parents.

## Discussion and conclusion

PALF is a rare but life-threatening illness with a high risk of progression to multiorgan failure and death [7]. The common causes of PALF include infection, toxins/drugs, genetic metabolic disorders, immune-mediated diseases, infiltrative diseases, and vascular/ischemic causes. Mushroom poisoning is a relatively rare cause of PALF. There are more than 3000 species of mushrooms in China. Approximately 400 of these are toxic to humans. Over 90% of fatal mushroom poisonings in the world occur after ingestion of *Amanita* species [8–10]. There are two distinct groups of toxins isolated from *Amanita*: phallotoxins and amatoxins. Although phallotoxins is highly toxic to hepatocytes, it is not the cause of liver failure, because phallotoxins are not absorbed from the gastrointestinal tract and do not reach the liver. Amatoxins are the main toxins that cause ALF which accounts for about 90% of fatalities [11]. Patients with amatoxin-induced ALF have a poor prognosis, and LT is the only lifesaving therapy. In the pretransplantation era, the survival rate was 10–30%; however, transplantation has greatly improved the survival rate to 87% [10, 12]. At present, reports on LT for PALF caused by mushroom poisoning are mainly from developed countries [13]. There are few reports about this topic in mainland China.

After the toxin enters the intestinal tract, amatoxin binds to DNA-dependent RNA polymerase II, inhibits protein synthesis, and induces the production of cytokines, ultimately resulting in the death of hepatocytes [7, 10]. The clinical course of amatoxin poisoning is classically divided into four consecutive phases. (1) Asymptomatic Phases. There are no symptoms and signs at this stage, and it lasts generally 6–40 h after ingestion with an average of about 10 h. (2) Gastrointestinal Phase. This phase is characterized by nausea, vomiting, abdominal pain, diarrhea, dehydration etc. It lasts for 12–24 h. At this time, the patient’s liver function and renal function are normal. If the doctor does not get the history of mushroom ingestion, it is easy to be misdiagnosed as gastroenteritis. (3) Latent Hepatic Phase. It occurs 36–48 h after ingestion. Although gastrointestinal symptoms are significantly improved, the performance of liver involvement is gradually obvious, jaundice, transaminases and bilirubin increase. Renal function is involved. (4) Acute Liver Failure Phase.This stage is characterized by progressive deterioration of liver function (hyperbilirubinemia, coagulation dysfunction, hepatic encephalopathy and hepatorenal syndrome). If liver failure progresses irreversibly, multiple organ failure and death usually occur within 1–3 weeks after ingestion [11].The spontaneous survival rate of patients with mushroom poisoning causing acute liver injury or ALF is between 33.8% and 55.6% [6]. ALF is characterized by apoptosis or necrosis of the parenchymal cells [14]. The clinical manifestations and pathology of this case are consistent with those of typical mushroom poisoning.

There is no specific antidote to amatoxin. The common therapies include gastric lavage, multidose activated charcoal, cimetidine, penicillin G, silybin, and N-acetylcysteine. Artificial liver support systems are also used in some centers. However, due to the lack of randomized controlled trials, these methods have not been proven effective [9, 12]. When these treatments fail, LT is the only lifesaving option. Although transplantation has substantially improved the short-term survival rate, long-term rates are still lower than those of other indications [14, 15]. This may be due to the lack of long-term follow-up data because of a relatively recently introduction of PLT. The liver has the potential for spontaneous recovery, and some patients with PALF can survive without LT. Therefore, the specific criteria and optimal timing of LT remain to be determined [15, 16].

Traditionally, transplant decisions are based on the 2 most recognized models: The KCH or Clichy criteria. The KCH criteria are the most widely used for urgent LT in patients with ALF and are currently the basis for emergency LT selection by the UK National Health Service Blood and Transplant. Patients are divided into two groups: paracetamol (PCM) and non-PCM groups. The parameters incorporated into the KCH criteria, which include HE, the INR, age, serum bilirubin, renal function, and acidosis, are considered to be prognostic markers for death in ALF patients [6]. Studies reporting KCH criteria have shown positive predictive values ranging from 70% to almost 100% and negative predictive values ranging from 25–94% [17]. The KCH criteria has been criticized for its low sensitivity and negative predictive value for a poor outcome. In the present study, the patient fulfilled 4 of 5 of the KCH criteria and met the criteria for LT. Vinay Sundaram utilized the PALF study group database and revealed that KCH did not reliably predict death in PALF patients. However, the study had limitations. It was a retrospective study, and supportive management may have affected laboratory values, such as the INR [18]. Therefore, KCH in patients with PALF still needs further validation in high-quality prospective studies. The Clichy criteria have been widely used in Northern Europe and consider age, HE, and factor V concentration. These criteria were predicted to have a positive predictive value of 82% and a negative predictive value of 98%. However, they were derived from an adult cohort with fulminant hepatitis B infection and thus cannot be generalized to all patients with ALF [17]. The Clichy criteria could not be applied to this case because we did not measure the factor V level in the patient. This is one of the study’s limitations [17].

The above two criteria were designed for adults, and their application in children is still controversial; therefore, we need updated standards for children. The PELD score and LIUs are useful for only pediatric patients. The PELD model evaluates 5 parameters: age, growth failure, bilirubin, ALB, and INR. The PELD score is used to aid the allocation and prioritization of LT in children (≤ 12 years old) with end-stage liver disease. It was developed to evaluate chronic liver disease rather than acute liver disease, and it considers some factors that are irrelevant to the prognosis of ALF, such as growth disorders. However, some studies have found it useful. Raquel Núñez-Ramos et al. revealed that PELD scores higher than 28 showed a specificity and a positive predictive value of 100% for identifying patients who will not recover spontaneously [14]. Rajanayagam J reported that PELD thresholds of ≥ 27 according to the PALF criteria and ≥ 42 at the peak predicted poor outcomes [19]. This case’s PELD score on the day of LT was 40, accordant with Raquel Núñez-Ramos’s but not accordant with Rajanayagam J’s research. Above all, PELD scores are still controversial in judging the timing of PLT. LIUs were devised from a single-center retrospective analysis of a cohort of PALF patients. This formula includes peak bilirubin, INR (or PT), and ammonia. It is stratified into low/moderate and high risk categories. It has high specificity and sensitivity for predicting death/LT [6]. It has been recommended by Chinese PALF guidelines. The LIUs of this patient was 242. He had a high risk for death/LT. The decision to consider only the LIUs for LT is insufficient, especially for LT due to different etiologies.

Escudie et al. and Ganzert et al. studied specific criteria to assess the need for transplantation in patients with amatoxin poisoning; however, these criteria for LT are not universally accepted [20]. The decision of LT for this patient took into account the rapid progression of the disease, poor response to treatment and high LIU score. In the future, it is expected that there will be an accurate prediction model for PALF due to mushroom poisoning.

In this study, we did not identify the causative mushroom due to the lack of laboratory equipment. In China, the identification of mushroom species consumed by patients is difficult in most hospitals [10]. The history of mushroom ingestion is essential for diagnosis.Therefore, the patient was suspected to have ingested amatoxin-containing mushrooms.

In conclusion, mushroom poisoning can induce PALF and result in a very poor prognosis without PLT. Early identification and diagnosis of PALF is important. It is necessary to transfer patients with PALF to a hospital with a LT center as early as possible. Once conservative treatment fails, LT should be carried out. At present, optimal prognostic criteria to predict death in PALF patients without LT are still lacking. In order to find the best timing for LT, the best solution is that clinicians with experience in PALF and LT continuously evaluate the possibility of spontaneous recovery in combination with etiology and response to treatment.

## Abbreviations

PALF: Pediatric acute liver failure
INR: International normal ratio
LT: Liver transplantation
PLT: Pediatric liver transplantation
KCH: King’s College Hospital
ALF: Acute liver failure
LIUs: Liver injury units
PELD: Pediatric End-Stage Liver Disease
ALT: Alanine aminotransferase
TBIL: T otal bilirubin
DBIL: Direct bilirubin
PT: P rothrombin time
GCS: Glasgow Coma Scale
HE: Hepatic encephalopathy
PCM: Paracetamol

## Availability of data and materials

The datasets generated and/or analysed during the current study are not publicly available due to the aim to protect the confidentiality of the family but are available from the corresponding author on reasonable request.
